# Effects of wilting and additives on the ensiling quality and *in vitro* rumen fermentation characteristics of sudangrass silage

**DOI:** 10.5713/ajas.20.0079

**Published:** 2020-06-23

**Authors:** Jiang Chun Wan, Kai Yun Xie, Yu Xiang Wang, Li Liu, Zhu Yu, Bing Wang

**Affiliations:** 1 College of Animal Science and Technology, China Agricultural University, Beijing 100193, China; 2 College of Grassland and Environment Science, Xinjiang Agricultural University, Urumqi, 830052, China

**Keywords:** Fermentative Profile, *In vitro* Rumen Fermentation, Wilting, Additives, Sudangrass

## Abstract

**Objective:**

This study was conducted to investigate the effects of molasses and *Lactobacillus plantarum* on the ensiling quality and *in vitro* rumen fermentation of sudangrass silage prepared with or without wilting.

**Methods:**

The ensiling experiment, measured with 3 replicates, was carried out according to a 2×4 (wilted stages×additives) factorial treatment structure. Dry matter of the fresh (210 g/kg fresh matter) or wilted (305 g/kg fresh matter) sudangrass were ensiled (packed into 5.0-L plastic jars) without additive (control) or with molasses (M), *Lactobacillus plantarum* (LP), or molasses + *Lactobacillus plantarum* (M+LP). After 60 days of ensiling, the silages were analyzed for the chemical, fermentation, and *in vitro* characteristics.

**Results:**

After 60 days of ensiling, the fermentation parameters were affected by wilted, the additives and the interactions of wilted with the additives (p<0.05). The M+LP treatment at wilted had higher lactic acid levels and V-score (p<0.05) but lower pH values and butyric acid concentrations than the other treatments. In comparison with sudangrass before ensiling, after ensiling had lower dry matter and higher non-fibrous carbohydrate. The *in vitro* gas production, *in vitro* dry matter digestibility, *in vitro* crude protein digestibility, and *in vitro* acid fiber detergent digestibility changed under the effects of the additives. Significant interactions were observed between wilted and the additives in terms of *in vitro* gas production at 48 h, asymptotic gas production, gas production rate, half time, and the average gas production rate. The total volatile fatty acid levels in the additive treatments were higher than those in the control.

**Conclusion:**

Wilting and supplementation with molasses and *Lactobacillus plantarum* had the ability to improve the ensiling quality and *in vitro* nutrient digestibility of sudangrass silage. The M+LP treatment at wilted exhibited the strongest positive effects on silage quality and *in vitro* ruminal fermentation characteristics.

## INTRODUCTION

Sudangrass (*Sorghum sudanense* (Piper) Stapf) is a major forage grass species that is widely cultivated in West China due to its high salt tolerance. Annually, more than 1,000 hectares of sudangrass are grown as a feed source. Ensiling is a good method by which sudangrass can be processed and preserved, especially in places where it always rains during the harvest period. However, the low dry matter (DM)(<280 g/kg DM) content of sudangrass results in poor silage quality. It has been reported that butyric acids (BA) are produced due to high moisture content during the ensiling of sudangrass [1]. It is known that in low-DM concentration silage some groups of *clostridia* can act by converting water soluble carbohydrate (WSC) into BA. Moreover, the high moisture concentration of silage produces a higher proteolytic activity by the *clostridium* proteolytic group. Often the poor fermentation quality of any grass silage is associated with high moisture and delay in pH drop because of the low WSC concentration. The poor fermentation characteristics limit the utilization of sudangrass as livestock feed, thus, effective measures, such as wilting, use of acid-type additives (e.g., potassium diformate, sodium diacetate), inoculation (e.g., homofermentative lactic acid bacteria, heterofermentative lactic acid bacteria, combination inoculants containing homofermentative and heterofermentative lactic acid bacteria) and use of enzymes (e.g., cellulase, hemicellulase) should be used to improve the ensiling quality.

The key measure to determine improvement of the ensiling quality is mainly lactic acid production during ensiling. Molasses is used extensively as a fermentation stimulant since this material provides fermentable substrates for lactic acid bacteria (LAB) [2]. Zhang et al [3] found that *Urtica cannabina* treated with molasses at either 4% or 8% produced well-preserved silage with a faster acidification rate and greater lactic acid (LA) levels than untreated silages. Kang et al [4] found that treatment of four-month cassava top with 2% molasses improved the silage quality and the *in vitro* rumen fermentation efficiency. Supplementation with homo-LAB leads to efficient WSC utilization, resulting in rapid reduction of pH and decreased nutrient loss. Many researchers [5,6] have focused on improvement of the fermentation quality of silage via inoculation with *Lactobacillus plantarum* (*L. plantarum*) because this microbe produces sufficient LA for rapid reduction of the pH. Wilting is an efficient, widely used pretreatment method in ensiling, with the ability to decrease plant protease activity, clostridial growth, and the amount of silage effluent, as well as to increase the sugar concentrations in crops, thereby improving the nutritional quality of the silage [7].

*In vitro* gas production (GP) is well-accepted technique for measurement of the ruminal degradation rates of feedstuffs. *In vitro* studies have detected increased microbial biomass yields caused by inoculation of silages with molasses and *L. plantarum* with different GP levels and total volatile fatty acid (VFA) concentrations [5]. A recent study demonstrated that inoculation of silage with *L. plantarum* elicits different *in vitro* responses [6]. In addition, the lag time of feedstuff has been reported to be affected by the rumen liquor. Consequently, *in vitro* GP and DM digestibility might also be affected. Either wilting or additives could improve fermentation quality and reduce nutrient loss during ensiling. To date, research on ensiling quality and *in vitro* ruminal fermentation characteristics of sudangrass silage with both wilting and additives is lacking, and whether the wilting and additives have positive effects on *in vitro* GP and DM digestibility on sudangrass silage is not clear. Thus, the aim of this study was to determine the effect of wilting and supplementation with molasses and *L. plantarum* on the quality of sudangrass silage.

## MATERIALS AND METHODS

### Sudangrass harvest

Pure sudangrass (*Sorghum sudanense* (Piper) Stapf) was grown in experimental plots at Sanping practice base in Xinjiang Agricultural University (latitude 43°54′N, longitude 87°19′E), which is situated in Xinjiang province, China, from April to July 2019, using Xinsu sudangrass variety No. 3 (Ministry of Education Key Laboratory for Western Arid Region Grassland Resources and Ecology, Xinjiang, China). Triplicate randomized experimental plots were used, each plot with an area of 5 m×10 m. Approximately 45 kg/hm^2^ of cow manure fertiliser being applied during the sudangrass growing. The soil type in this area is typically loamy soil and the average soil organic matter in the experimental plots was 15.6 g/kg; effective N, P, and K in the soil were 91.3, 23.7, and 103.1 mg/kg, respectively. The sudangrass was sown in 25 April 2019 at a seeding rate of 105 kg/hm^2^ in each block and the first-cut sudangrass was manually harvested in 6 July 2019 by sickle at the early-heading stage from three replicated plots. The plant materials were chopped to a 20-mm nominal length and then sampled in triplicate and stored at −20°C prior to chemical analyses.

### Experimental design and silage preparation

The ensiling experiment was carried out according to a 2×4 (wilted stages×additives) factorial treatment structure. The sudangrass was direct wilted at the field in the sun for 8 h (direct-cut sudangrass) and turning of the forage every 2 h. The factors associated with the wilted stage were fresh or wilted (8 h), and the 4 additive treatments were i) no additive (control, CK); ii) molasses (M) (Tianshun Bioengineering Co., Ltd., Urumqi, China) applied at 5% fresh weight (FW); iii) *L. plantarum* (LP) (strain 10474, China General Microbiological Culture Collection Center, Beijing, China) applied at 1×10^5^ colony forming units (CFU)/g FW; iv) molasses (M, Tianshun Bioengineering Co., Ltd, Urumqi, China) applied at 5% FW + *L. plantarum* (LP, strain 10474, China General Microbiological Culture Collection Center, Beijing, China) applied at 1×10^5^ CFU/g FW. Based on the manufacturer’s information, the molasses contained 45.2% moisture, 59.2% total sugar DM, 38.4% sucrose and 12.4% reducing sugars. According to the set additive amount, the molasses, *L. plantarum*, and molasses + *L. plantarum* were diluted to 0.5 L with water, respectively. The same amount of water was added to the control. The additives were diluted in deionized water and each treatment was sprayed in a fine mist on the sudangrass from each of the appropriate replicate plots separately using a hand sprayer (Sail Multipurpose sprayer, Sail Corp., Beijing, China). Hand forks were used to mix the forage from each treatment. The materials (3,200 g) were individually packed into 5.0-L plastic jars (Hewanglan Paper and Plastic Products Factory, Beijing, China) and sealed with plastic tape and caps. Each treatment stored at an ambient temperature of approximately 26.0°C±3.7°C. After 60 days of ensiling, the jars were opened and sampled; the samples were stored at −20°C for subsequent chemical analysis of the DM, crude protein (CP), neutral detergent fiber (NDF), acid detergent fiber (ADF), ether extract (EE), ash, pH, LA, acetic acid (AA), propionic acid (PA), BA, WSC, NH_3_-N (ammonia nitrogen), and *in vitro* batch cultures.

### Chemical analyses

Following the method described by Owens et al [8], 20 g of each fresh silage sample was homogenized in a blender with 180 mL of distilled water for 1 min and then filtered through four layers of cheesecloth. Then, the pH of the filtrate was measured immediately using a glass electrode pH meter (PHS-3C, INESA Scientific Instrument Co., Ltd., Shanghai, China). The DM content was determined by oven drying at 60°C for 48 h. The DM recovery was calculated as (1 – [(ensiled forage DM – forage DM at silo opening)/ensiled forage DM])×100. NDF and ADF levels were measured as described by Mertens [9]. The ash content was determined by ignition at 550°C for 3 h, and the EE was examined according to the methods described by the Association of Official Analytical Chemists (AOAC) [10]. The buffering capacity (BC) of the fresh matter (FM) was determined by titration [11]. The WSC levels were determined using the anthrone method [12]. The nitrogen (N) content was analyzed according to the methods of the AOAC [10], and the CP content was estimated by multiplying the N content by 6.25.

The pH values of the *in vitro* ruminal fluid samples were measured using a pH meter as described above. The NH_3_-N concentrations in the silage were determined by the method described by Broderick and Kang [13] and expressed as g/kg of total nitrogen (TN). The levels of organic acids, including lactic acid, AA, PA, and BA, in the silage and *in vitro* ruminal fluid samples were determined by high-performance liquid chromatography (LC-20A; Shimadzu, Tokyo, Japan). The analytical conditions were as follows: column, Shodex RSpak KC-811S-DVB gel C (8.0 mm×30 cm, Shimadzu, Japan); oven temperature, 50°C; mobile phase, 3 mmol/L HClO4; flow rate, 1.0 mL/min; injection volume 5 μL; detector, SPD-M20AVP (Shimadzu, Japan). To assess the ensiling quality, the V-score from the NH_3_-N and VFA concentrations were calculated [14].

### Microbiological analyses

The LAB counts were determined using MRS agar and violet red bile agar after incubation at 30°C for 2 days. Aerobic bacterial counts were determined after growth on nutrient agar at 37°C for 3 days under aerobic conditions. All yeasts and moulds were enumerated on spread plates containing yeast extract/peptone/dextrose agar and salt Czapek Dox agar, respectively, after incubation at 28°C for 3 to 5 days. The four media were obtained from Beijing Aoboxing Biotech (Beijing, China). All microbial data were transformed to log10 values and presented on a wet weight basis.

### *In vitro* rumen fermentation

Silage samples were oven-dried at 65°C, ground, and sieved through a 1-mm screen. The dried silages (500 mg) were weighed into 120-mL glass bottles with butyl rubber stoppers and Hungate’s screw caps. Five bottles per silage sample and 30 bottles per treatment were arranged. Fifty milliliters of a freshly prepared buffer solution (pH 6.85) and 25 mL of filtered rumen fluid collected from three rumen-fistulated lactating Holstein dairy cows (423±18 kg body weight, the animals were restricted to a supply of the same amount of a standardized diet containing 5.0 kg of alfalfa hay, 2.0 kg of wheat straw and 6.0 kg of commercial concentrate supplement per day) were added to the bottles before the morning feeding. The bottles were purged anaerobically with CO_2_ for 5 s, sealed with the butyl rubber stopper and Hungate’s screw caps, and individually connected with medical plastic infusion pipes to gas inlets of an automated trace gas recording system to continuously record cumulative GP (0, 2, 4, 8, 12, 24, 36, 48 h). All bottles were incubated at 39°C for 48 h, and the entire experiment was repeated for three runs. At the end of incubation, the culture fluids in each bottle were individually filtered with pre-weighed nylon bags (8×12 cm, 42-μm pore size). The pH value, NH_3_-N content and organic acid content of the filtrate were determined according to the method described above. The bags were thoroughly rinsed and dried at 65°C for 48 h to a constant weight. The differences between DM, CP, NDF, and ADF at initial incubation and the residual DM, CP, NDF, and ADF, respectively, corrected with the blanks after incubation were calculated to determine the *in vitro* DM, CP, NDF, and ADF disappearance (IVDMD, IVCPD, IVNDFD, and IVADFD, respectively) values.

### Biometric analysis

The cumulative GP (*t*, mL/g DM) at time (*t*) for each fermentation bottle was fitted to an exponential model [15]:

(1) $${\text{GP}}_{\text{t}}={\text{A}}^{\prime }[1-{\text{e}}^{-{\text{c}}^{\prime }(\text{t}-\text{Lag})}]$$

where ‘e’ is the base of a natural logarithm; A represents the asymptotic GP generated at a constant fractional rate (*c*) per unit time; *t* is the gas recording time, and Lag represents a lag time phase before commencement of GP. The average GP rate (AGPR, mL/h) was defined as the rate between the start of incubation and the time at which the GP was half of its asymptotic value according to the following equation [16]:

(2) $$\text{AGPR}=\frac{A\times c}{2\times (Ln2+c\times Lag)}$$

The ratio of non-glucogenic to glucogenic acids (NGR) was calculated as described by Orskov [17] as follows:

(3) $$\text{NGR}=\frac{Acetate+2\times Butyrate+Valerate}{Propionate+Valerate}$$

where acetate, propionate, butyrate and valerate were expressed in molar proportions.

The fermentative characteristics, microbial counts, and *in vitro* and chemical compositional parameters were analyzed for significant differences via analysis of variance (ANOVA), with significance reported at a probability level of 0.05, using the general linear model in SPSS (version 21.0, SPSS Inc., Chicago, IL, USA). All data were subjected to ANOVA using the general linear model procedure in SPSS, based on the following model:

$${\text{Y}}_{\text{ij}}=\mu +{\text{W}}_{\text{i}}+{\text{A}}_{\text{j}}+{\left(\text{W}\times \text{A}\right)}_{\text{ij}}+{\text{e}}_{\text{ij}}$$

where Y_ij_ is the response variable (e.g., GP, fermentation kinetics parameters); μ is the overall mean; W_i_ is the wilted stage of the sudangrass (i = fresh or wilted); A_j_ is the additive (j = control, molasses, *L. plantarum* or molasses + *L. plantarum*); (W×A)_ij_ is the interaction term between wilted and additives; and e_ij_ is the residual error. Differences between treatment means were compared using the least square means method and Tukey’s multiple comparison test. The results are reported as least square means and the associated standard errors.

## RESULTS

### Chemical and microbial compositions of materials before ensiling

The included as a chemical composition of the sudangrass before ensiling are presented in Table 1. The DM, ADF, and ash content of the sudangrass after wilting was higher than that fresh. In contrast, the WSC and BC content of the sudangrass after wilting was lower than that fresh. The fermentation coefficient was lower than 25 for both treatments. The number of epiphytic LAB on the sudangrass was less than 1.0×10^5^ CFU/g FM, and the number of yeast cells was approximately 1.0×10^3^ CFU/g FM.

### Fermentation quality of sudangrass silage after 60 days of ensiling

The six sudangrass silages were well preserved with additives, as indicated by the low pH value and NH_3_-N proportion and high LA content and V-score compared to the control (Table 2). The pH, LA, AA, PA, BA, WSC, NH_3_-N levels and V-score were significantly influenced by wilted, the additives, and the interaction of wilted with the additives (p<0.05). No significant interaction between wilted and additives was observed in terms of the counts of yeasts (p = 0.150) and moulds (p = 0.158). The BA content was less than 5.0 g/kg DM in all the treatments except the control of the fresh. Except for the control of the fresh, all the silages were fermented, with the V-score in all treatments ranging from 51 to 89 after 60 days of ensiling. The M+LP treatment at wilted exhibited significantly increased LA content and V-score and decreased pH and BA values compared to the other treatments.

The chemical composition of the silages is listed in Table 3. After silage, the DM, DM recovery, ADF, and ash levels were higher at wilted than fresh. The NDF of the wilted-treated was not significantly different from that of the fresh-treated. The additive-treated groups had lower ADF levels than the control in the wilted-treated (p<0.05), however, there was no significant difference of effects of wilted, additives and interaction between wilted and the additives on CP. The interaction between wilted and the additives was significant for EE (p<0.001), ash (p<0.001) and non-fibrous carbohydrate (NFC) (p = 0.013). At fresh, after silage-treated had lower DM and CP contents and higher NFC than the before silage, there was no significant difference for NDF before and after the fermentation of silage. At wilted, after ensiling had lower DM contents and higher NFC than before ensiling, in contrast, there was no significance difference of CP before and after ensiling.

### *In vitro* gas production

**Figure 1:**
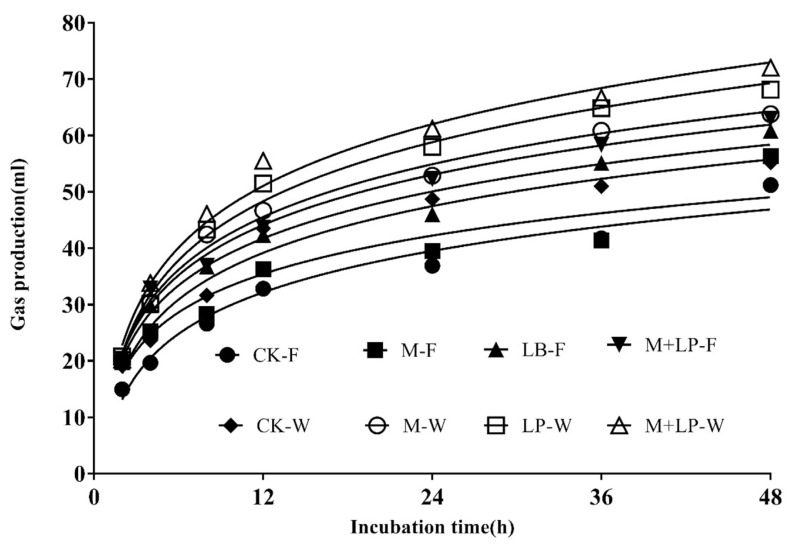
Gas production profiles from *in vitro* fermentation of sudangrass silages for 48 h. C-F = control-Fresh; M-F = molasses-Fresh; LP-F = *L. plantarum*-Fresh; M+LP-F = molasses+*L. plantarum*-Fresh; C-W = control-Wilted; M-W = molasses-Wilted; LP-W = *L. plantarum*-Wilted; M+LP-F = molasses+*L. plantarum*-Wilted.

The GP profiles of the sudangrass silages are presented in Figure 1. Significant differences were observed among treatments in terms of gas volume. As shown in Table 4, the IVADFD values were higher in additive-treated silages than in the control. Significant interactions were observed between wilted and additives in terms of *in vitro* GP (p<0.001) at 48 h of incubation. In the Kinetic PG model, significant interactions were observed between wilted and additives of the asymptotic GP (A) (p<0.001), GP rate (p = 0.005), half time (p<0.001) and AGPR (p<0.001), however, there was no effect on lag time (p = 0.341). The total 48-h GP was increased by supplementation with additives and was higher than in the control. The estimated values for A were in highest for the M+LP silage at wilted.

The NH_3_-N, NGR, and total VFA levels were affected by wilted, the additives and the interaction of wilted with the additives (p<0.05; Table 5). The total VFA content in the additive treatments was higher than that in the control. All the additive treatments decreased the pH levels compared to the control. The highest total VFA content (115 mmol/L) was observed in the LP treatment at wilted. The interaction between wilted and the additives affected the VFA pattern, including the concentrations of acetate (p = 0.023), propionate (p<0.001), butyrate (p<0.001), isobutyrate (p = 0.022), valerate (p<0.001), and isovalerate (p<0.001).

## DISCUSSION

### Chemical and microbial compositions of materials before ensiling

The most significant change of forage chemical composition caused by wilting is to reduce the water content. Low water content can effectively inhibit the fermentation of *clostridium*, to improve the smell of silage and effectively inhibit the occurrence of mildew. The DM content of grass strongly influences the rate and extent of ensiling. Grasses with a low DM and sugar levels at pre-ensiling may exhibit clostridial fermentation and subsequent poor acceptance of the silage by animals [18]. McDonald wrote that wilting can increase the content of the DM and WSC [7], however, similar results were not observed in this experiment. This may be due to the high lignification of the sudangrass stalk, which is difficult to break during processing, and with the loss of the water the content of WSC will also decreased. According to McDonald [7], a material can be considered to be successfully ensiled if it exhibits favorable DM levels (280 to 340 g/kg DM), fermentable sugar concentrations ranging from 30 to 50 g/kg DM, a low BC and LAB abundances greater than 1×10^5^ CFU/g FM. In this study, the DM of the sudangrass in fresh before ensiling was lower than this standard, suggesting that it might be difficult to obtain high-quality silage by direct ensiling of sudangrass.

### Fermentation quality of sudangrass silages

Silage pH is one of the main factors that influences the extent of fermentation and ensiling quality [19]. The low final pH (4.70 or less) in the wilted and additive-treated sudangrass silages suggested the recommended standard for high-quality silage (4.30 to 4.70) [20]. As expected, wilted sudangrass silage supplemented with *L. plantarum* improved the LA levels under the increased the microorganism concentration. After wilting, the sudangrass silages supplemented with additives were all well preserved and exhibited moderate fermentation quality, despite the low WSC content and numbers of epiphytic LAB in the initial herbage. The fermentation of the wilted silage exhibited a higher target ensiling DM due to elevated total VFA and LA concentrations. The ensiling quality at wilted is usually better than that fresh [21], which is consistent with the results of this study. The presence of BA in silage is undesirable given that its generation is an energy-waste metabolism and BA>5 g/kg DM is an indicator of substantial clostridial activity reducing feed intake and causing health issues [22]. In the present study, the high BA content (12.3 g/kg DM) indicated extensive clostridial fermentation in control-treated fresh silage. Wilting and additives could help to inhibit the activity of undesirable organisms in silage. It is supported by the decrease of BA content, where BA were not higher than 5 g/kg DM in those treatments. In addition, wilting and *L. plantarum* can reduce the content of AA and BA, which can improve palatability of the silage. Similar results have been reported by Zhang et al [23], who found wilting and *L. plantarum* decreased AA and BA contents of mulberry silage.

NH_3_-N production reflects the extent of proteolysis in silage. Well-preserved silages should have less than 100 g/kg TN [22]. In the present study, the value of NH_3_-N was less than 94.2 g/kg TN at wilted treatment, indicating that extensive proteolysis may not have occurred. Microorganisms exhibit proteolytic activity mainly in low-DM silages at high pH values (i.e., >4.2) when the growth and activity of the microorganisms is not totally inhibited under such conditions [7], which may partly explain why the NH_3_-N levels were lower at wilted than fresh (higher DM and lower pH values were observed at wilted than fresh).

Molasses and the LAB inoculants used in this study are commonly used to improve the fermentation quality of sudangrass silage. Furthermore, the increased LAB counts and decreased mould counts after supplementation with additives suggest that high-quality silage was obtained, which might considerably improve the dry matter intake (DMI) of ruminants. It is well known that adequate WSC levels and LAB counts are important for rapid establishment and growth of LAB. Molasses were added to provide the WSC levels necessary for ensiling fermentation. Microbial inoculation, especially of homofermentative LAB, is a common practice for acceleration of fermentation, resulting in high-quality silage and reduced DM loss. Adesogan et al [24] found that addition of exogenous *L. plantarum* accelerated homofermentation, allowing increased production of LA by *L. plantarum*. Thus, the increased LA content in the M+LP treatment at wilted might be attributed mainly to using molasses together with *L. plantarum*, which improved the WSC and LAB content before ensiling. Muck et al [25] concluded that microbial inoculation lowered the pH and improved the LA content, and Jahanzad et al [26] confirmed that molasses can enhance the activity of homofermentative bacteria, which convert WSC to LA in millet and soybean silage. *L. plantarum*, used for inoculation in this study, is a homofermentative lactobacillus that can ferment a wide variety of substrates and rapidly produce large amounts of lactic acid. *L. plantarum* had positive effects on grass silage fermentation characteristics by lowering the pH and shifting fermentation towards LA, which is consistent with the results of this study.

From a practical standpoint, wilting can reduce risk and increase DM levels during ensiling, which can be used to improve silage quality. The results in this study demonstrated that wilting is an effective measure.

### *In vitro* gas production

*In vitro* GP is associated with the availability of NFC and fermentable carbohydrate content from the substrate. Evaluation of *in vitro* GP is also an indicator of rumen digestion, as *in vitro* GP affects the rumen passage rate and DMI. In this study, the IVDMD was higher in M+LP treatment at wilted than in other groups, which might be attributed to the decreased DM loss in silage after supplementation with molasses and *L. plantarum*. Thus, the IVDMD of the rumen was elevated. Additionally, the M+LP treatment at wilted provided and preserved the WSC and LA content in silage, which further benefited *in vitro* rumen degradation. In the present study, the potential GP, A and GP rates of additive-treated silages were higher than those of the control, probably due to the additive-treated reduced loss of nutrients after additive supplementation.

GP has been reported to be positively correlated with VFA production. The total VFA concentrations were significant difference between the control and the M+LP-treated silage at wilted at 48 h of fermentation. GP is a direct result of microbial degradation of feed and an indirect result of the buffering of acids generated by fermentation [27]. Moreover, additional gas is produced simply by contact between acids (VFAs and LA) produced in the silo and the buffer inoculum. Therefore, indirect GP from acids produced in the jars and during *in vitro* fermentation might explain the positive relationship between GP and VFA concentration in the present study.

The total VFA is one of the main energy sources for ruminant growth and development, which provides about 70% to 80% of the energy required by ruminants [28]. Although the composition and concentration of VFA in rumen were affected by feed concentrate/roughage ratio, processing method and additives, the highest content of VFA in rumen was the acetate [29]. The present study had similar results, which supports the previous studies. The VFA pattern o*f in vitro* rumen fermentation was affected by wilted, the additives and the interactions between wilted and the additives in the present study. *In vitro* ruminal fermentation, the treatment after wilting and additives, affected the contents of AA, PA, and BA so that they were increased in comparison with the control. It could be explained that wilting and additives can improve the fermentation quality of sudangrass silage, especially the content of lactic acid, which can be degraded into acetate, propionate, and butyrate [30]. Thus, the VFA content of the rumen was elevated.

## CONCLUSION

It was concluded that wilting and supplementation with molasses and *L. plantarum* affected the ensiling quality and *in vitro* ruminal fermentation characteristics of sudangrass silage. In comparison with sudangrass before ensiling, after ensiling had lower DM and higher NFC. Supplementation with molasses and *L. plantarum* prevented accumulation of BA and resulted in increased homolactic acid fermentation. The *in vitro* rumen digestibility of silage can be regulated by wilted and additive treatment. The best treatment for sudangrass silage was M+LP at wilted, based on ensiling quality and *in vitro* ruminal fermentation characteristics.

## Figures and Tables

**Table 1:** Chemical composition and microbial populations contents of sudangrass before ensiling

Items	Treatment		SEM	p-value

	Fresh	Wilted		
Dry matter (g/kg FM)	210	305	21.4	<0.001
Water soluble carbohydrate (g/kg DM)	87.7	52.2	8.01	<0.001
Crude protein (g/kg DM)	134	115	5.37	0.077
Neutral detergent fibre (g/kg DM)	647	631	4.76	0.095
Acid detergent fibre (g/kg DM)	344	370	6.13	0.006
Ether extract (g/kg DM)	29.4	29.3	0.34	0.901
Ash (g/kg DM)	60.4	72.1	2.62	<0.001
NFC1) (g/kg DM)	129	152	6.46	0.065
Buffering capacity (mEq/kg DM)	249	162	20.5	0.004
Fermentation coefficient2)	19	24	0.01	0.001
pH	5.37	5.17	0.06	0.071
Lactic acid bacteria (log_10_ CFU/g FM)	4.67	3.62	0.24	<0.001
Aerobic bacteria (log_10_ CFU/g FM)	4.87	4.75	0.05	0.315
Yeasts (log_10_ CFU/g FM)	3.26	2.90	0.08	0.002
Moulds (log_10_ CFU/g FM)	6.14	5.78	0.09	0.008

SEM, standard error of the mean; FM, fresh matter; DM, dry matter; NFC, non-fibrous carbohydrate; mEq, milligram equivalent; CFU, colony-forming units.

1) NFC = 100–CP–NDF–EE–ash.

2) Fermentation coefficient = DM (%) + 8 WSC/BC.

**Table 2:** Effect of wilting and additives on fermentation quality and microbial populations of sudangrass silage after 60 d of ensiling

Items	Treatments1)								SEM	p-value		

	Fresh				Wilted							
		
	CK	M	LP	M+LP	CK	M	LP	M+LP		Wilted	Additives	Wilted ×additives
pH	4.81^a^	4.50^c^	4.47^c^	4.36^d^	4.70^b^	4.28^e^	4.19^f^	4.06^g^	0.05	<0.001	<0.001	<0.001
Lactic acid (g/kg DM)	32.2^f^	46.2^e^	46.1^e^	50.5^d^	28.8^g^	55.1^c^	59.5^b^	66.0^a^	2.59	<0.001	<0.001	<0.001
Acetic acid (g/kg DM)	22.1^a^	8.68^c^	8.03^d^	6.23^f^	10.7^b^	7.14^e^	5.00^g^	4.69^g^	1.10	<0.001	<0.001	<0.001
Propionic acid (g/kg DM)	1.30^a^	0.86^b^	0.68^c^	0.65^cd^	0.89^b^	0.57^e^	0.59^de^	0.59^de^	0.05	<0.001	<0.001	<0.001
Butyric acid (g/kg DM)	12.3^a^	4.24^b^	4.57^b^	3.76^c^	4.24^b^	2.20^d^	1.89^d^	1.01^e^	0.68	<0.001	<0.001	<0.001
Water soluble carbohydrate (g/kg DM)	12.1^d^	18.1^a^	13.2^c^	17.2^b^	9.13^f^	13.1^c^	11.3^e^	13.0^c^	0.58	<0.001	<0.001	0.002
NH_3_-N (g/kg TN)	133^a^	74.6^c^	60.3^e^	65.5^d^	94.2^b^	65.6^d^	40.2^f^	41.6^f^	5.91	<0.001	<0.001	<0.001
Lactic acid bacteria (log_10_ CFU/g FM)	4.58^g^	6.13^e^	6.18^e^	7.38^c^	5.11^f^	6.54^d^	7.77^b^	8.56^a^	0.26	<0.001	<0.001	<0.001
Aerobic bacteria (log_10_ CFU/g FM)	3.20^b^	2.19^f^	2.42^e^	2.58^d^	3.49^a^	3.15^b^	2.88^c^	2.57^d^	0.09	<0.001	<0.001	<0.001
Yeasts (log_10_ CFU/g FM)	5.06^ab^	4.33^b^	4.58^ab^	4.49^ab^	4.90^ab^	5.30^a^	4.81^ab^	4.49^ab^	0.10	0.152	0.280	0.150
Moulds (log_10_ CFU/g FM)	4.18^a^	2.71^cd^	2.24^d^	3.49abc	3.89^ab^	3.08bcd	2.90bcd	2.70^cd^	0.16	0.961	0.003	0.158
V-score	27^g^	55^e^	56^e^	63^d^	51^f^	75^c^	82^b^	89^a^	3.88	<0.001	<0.001	0.001

SEM, standard error of the mean; DM, dry matter; NH_3_-N, ammonia nitrogen; TN, total nitrogen; CFU, colony-forming units; FM, fresh matter.

1) CK, control; M, molasses; LP, *L. plantarum*, M+LP, molasses+*L. plantarum*.

a–g Means within the same row with different superscripts differ significantly from each other (p<0.05).

**Table 3:** Chemical composition of sudangrass silages after 60 days of ensiling

Items	Treatments1)								SEM	p-value		

	Fresh				Wilted							
		
	CK	M	LP	M+LP	CK	M	LP	M+LP		Wilted	Additives	Wilted ×additives
Dry matter (g/kg FW)	193^cd^	196^c^	194^d^	191^d^	289^a^	286^a^	283^ab^	281^b^	9.83	<0.001	0.753	0.638
Dry matter recovery (%)	94.7^c^	94.6^c^	94.3^c^	93.5^c^	99.3^a^	98.1^ab^	98.0^ab^	97.1^b^	0.45	<0.001	0.233	0.090
Crude protein (g/kg DM)	111	109	108	114	108	107	113	113	0.92	0.817	0.189	0.365
Neutral detergent fibre (g/kg DM)	638^c^	647^ab^	640^bc^	635^c^	637^c^	643abc	648^a^	637^c^	1.17	0.544	0.002	0.087
Acid detergent fibre (g/kg DM)	356cde	353^de^	355cde	347^e^	375^a^	367^ab^	365^bc^	362bcd	2.02	<0.001	0.023	0.549
Ether extract (g/kg DM)	25.1^cd^	29.4^a^	26.0^cd^	24.8^d^	29.3^a^	25.4^cd^	27.4^b^	26.2^bc^	0.38	0.022	0.001	<0.001
Ash (g/kg DM)	65.6^c^	65.6^c^	65.1^c^	59.5^d^	70.9^a^	69.1^b^	71.4^a^	71.4^a^	0.82	<0.001	<0.001	<0.001
NFC (g/kg DM)	226^a^	214^bc^	226^a^	226^a^	227^a^	225^a^	212^c^	224^ab^	1.49	0.596	0.106	0.013

SEM, standard error of the mean; FW, fresh weight; DM, dry matter; NFC, non-fibrous carbohydrate.

1) CK, control; M, molasses; LP, *L. plantarum*, M+LP, molasses+*L. plantarum*.

a–e Means within the same row with different superscripts differ significantly from each other (p<0.05).

**Table 4:** *In vitro* rumen digestibility and gas production kinetics in culture fluids after 48 h incubation with different moisture content and additives of sudangrass silage

Items	Treatments1)								SEM	p-value		

	Fresh				Wilted							
		
	CK	M	LP	M+LP	CK	M	LP	M+LP		Wilted	Additives	Wilted ×additives
IVDMD	0.64^e^	0.65^de^	0.67^cd^	0.66cde	0.66cde	0.69^bc^	0.70^b^	0.73^a^	0.01	<0.001	0.001	0.124
IVCPD	0.62^bc^	0.60^cd^	0.61bcd	0.64^ab^	0.59^d^	0.61^cd^	0.65^a^	0.65^a^	0.01	0.351	<0.001	0.007
IVNDFD	0.51^d^	0.61^a^	0.56^bc^	0.54^c^	0.60^a^	0.54^c^	0.58^ab^	0.60^a^	0.01	0.003	0.199	<0.001
IVADFD	0.29^e^	0.36abc	0.37^ab^	0.38^a^	0.29^e^	0.33^cd^	0.31^de^	0.34bcd	0.01	<0.001	<0.001	0.085
Gas production (mL/g DM)	51.2^f^	56.5^e^	61.0^d^	63.1^c^	55.4^e^	63.7^c^	68.4^b^	72.0^a^	1.35	<0.001	<0.001	<0.001
Gas production kinetic												
A (mL)	51.2^f^	56.3^e^	60.8^d^	62.9^c^	55.2^e^	64.1^c^	68.2^b^	72.1^a^	1.36	<0.001	<0.001	<0.001
Gas production rate (/h)	0.26^f^	0.29^e^	0.32^d^	0.33^c^	0.29^e^	0.33^c^	0.36^b^	0.38^a^	0.01	<0.001	<0.001	0.005
Lag (h)	0	−0.01	0.01	−0.01	−0.02	0.01	0.01	0.01	0.00	0.681	0.254	0.341
Half time (h)	4.58^b^	2.95^f^	3.84^d^	3.57^e^	5.10^a^	4.04^c^	4.14^c^	2.58^g^	0.16	<0.001	<0.001	<0.001
AGPR (mL/h)	9.77^f^	12.0^e^	13.9^d^	15.0^c^	12.0^e^	15.4^c^	17.7^b^	19.5^a^	0.63	<0.001	<0.001	<0.001

SEM, standard error of the mean; IVDMD, *in vitro* dry matter digestibility; IVCPD, *in vitro* crude protein digestibility; IVNDFD, *in vitro* neutral detergent fibre digestibility; IVADFD, *in vitro* acid detergent fibre digestibility; DM, dry matter; A, the asymptotic gas production; Lag, the initial delay time in the onset of gas production; AGPR, the average gas production rate.

1) CK, control; M, molasses; LP, *L. plantarum*, M+LP, molasses+*L. plantarum*.

a–g Means within the same row with different superscripts differ significantly from each other (p<0.05).

**Table 5:** *In vitro* ruminal fermentation characteristics with different moisture content and additives of sudangrass silage

Items	Treatments1)								SEM	p-value		

	Fresh				Wilted							
		
	CK	M	LP	M+LP	CK	M	LP	M+LP		Wilted	Additives	Wilted ×additives
pH	6.81^a^	6.79^ab^	6.78^ab^	6.74^bc^	6.78^ab^	6.77^ab^	6.74^bc^	6.70^c^	0.01	0.008	0.002	0.732
NH_3_-N (mmol/L)	11.4^b^	10.5^c^	10.2^cd^	10.3^cd^	10.1^d^	11.2^b^	12.2^a^	10.3^cd^	0.15	<0.001	<0.001	<0.001
NGR	5.13^a^	4.92^a^	5.15^a^	4.52^b^	4.58^b^	4.12^c^	4.06^c^	4.47^b^	0.08	<0.001	0.001	<0.001
Total VFA (mmol/L)	98.0^f^	101^e^	106^c^	103cde	104^cd^	109^b^	115^a^	112^ab^	1.05	<0.001	<0.001	<0.001
VFA pattern (mol/100 mL)												
Acetate	67.3^d^	69.5^c^	69.4^c^	72.5^b^	69.3^c^	69.0^c^	71.5^b^	75.3^a^	0.51	<0.001	<0.001	0.023
Propionate	15.3^e^	16.6^d^	16.9^d^	18.6^c^	18.1^c^	18.1^c^	20.9^b^	22.2^a^	0.45	<0.001	<0.001	<0.001
Butyrate	8.91^e^	9.24^d^	9.96^bc^	10.2abc	8.56^f^	9.91^c^	10.3^ab^	10.4^a^	0.14	0.011	<0.001	<0.001
Isobutyrate	1.82^bc^	1.70^cd^	1.93^ab^	1.41^e^	2.07^a^	1.94^ab^	1.87^b^	1.61^d^	0.04	<0.001	<0.001	0.022
Valerate	1.68^c^	1.58^d^	1.48^e^	1.44^e^	1.77^bc^	1.85^b^	2.00^a^	1.52^de^	0.04	<0.001	<0.001	<0.001
Isovalerate	2.98^c^	2.60^d^	2.59^de^	2.51^e^	3.00^c^	2.99^c^	3.11^b^	3.22^a^	0.05	<0.001	<0.001	<0.001

SEM, standard error of the mean; NGR, ratio of non-glucogenic to glucogenic acids; VFA, volatile fatty acids.

1) CK, control; M, molasses; LP, *L. plantarum*, M+LP, molasses+*L. plantarum*.

a–f Means within the same row with different superscripts differ significantly from each other (p<0.05).
